# The Protective Effect of Antioxidants in Areca Nut Extract-Induced Oral Carcinogenesis

**DOI:** 10.31557/APJCP.2020.21.8.2447

**Published:** 2020-08

**Authors:** Dokyeong Kim, Rasika Pawiththra Illeperuma, Jin Kim

**Affiliations:** 1 *Department of Dental hygiene, Jeonju Kijeon College, Jeonju, Republic of Korea. *; 2 *Department of Medical Laboratory Science, Faculty of Allied Health Sciences, University of Peradeniya, Peradeniya, Sri Lanka. *; 3 *Department of Oral pathology, Oral Cancer Research Institute, Yonsei University College of Dentistry, Seoul, Republic of Korea. *

**Keywords:** Areca nuts, interleukin-6(IL-6), epithelial-mesenchymal transition(EMT), carcinogenesis, antioxidants

## Abstract

**Objective:**

Oral submucous fibrosis (OSF) is the premalignant disorder associated with fibrosis and epithelial atrophy. Areca Nut (AN) is the most significant risk factors for OSF. However, the molecular mechanism behind AN induced OSF remains unclear, and there exists no effective treatment for the malignant disorder. We aimed to investigate whether AN-extract causes epithelial-mesenchymal transition (EMT) in oral keratinocytes, and evaluated the therapeutic potential of antioxidants.

**Methods:**

The HPV16 E6/E7-transfected immortalized human oral keratinocytes (IHOK) were employed in the present study. For the preparation of AN-extract, dried AN was dissolved in distilled water overnight. The solution was centrifuged and the supernatant was collected for further use. For the determination of change in cytokine levels, ELISA was performed. To investigate EMT-related protein expression and phenotype, immunoblot and immunofluorescence were performed.

**Results:**

Among tumor-promoting cytokines (Gro-α, IL-6 and IL-8), IL-6 was remarkably increased by AN in IHOK. AN-extract induced EMT phenotypes, such as cell elongation, up-regulation of vimentin and snail. After treatment with neutralizing antibody of IL-6, AN-induced snail expression was reduced remarkably. Collectively, AN-extract induced *IL-6* expression and mediated EMT. The use of antioxidants (EGCG, glutathione and NAC) significantly reduced *IL-6* expression in AN-treated IHOK. Also, AN-decreased E-cadherin and increased vimentin were reversed by antioxidants, indicating that the effectiveness of antioxidants in inhibiting IL-6-induced EMT by AN.

**Conclusion:**

AN promotes EMT and antioxidants interrupt AN-induced-EMT in oral keratinocytes. Consequently, it is proposed that antioxidants could prevent AN-induced carcinogenesis and function as a prototype for developing therapeutic interventions of OSF.

## Introduction

Oral submucous fibrosis (OSF) is one of the potentially malignant disorders characterized by progressive fibrosis with epithelial atrophy in the oral cavity. OSF has been frequently reported in South Asia and parts of East Africa (Tilakaratne et al., 2006). About 7%-13% of them eventually transformed into oral squamous cell carcinoma (OSCC) (Chaturvedi et al., 2013; Arakeri et al., 2017). 

Areca Nuts (AN) are considered as the most significant risk factor for OSF. AN has been classified to independent group I human carcinogen by the International Agency for Research on Cancer (IARC) (Humans, 2004). Nevertheless, the molecular mechanism by which AN induces OSF and OSCC is still unclear. Furthermore, existing treatments for OSF provide only temporary symptomatic relief and until data, no effective treatment has been established. 

We previously suggested a molecular mechanism that transforms OSF to oral malignancy and the therapeutic potential of antioxidants in AN-induced oral carcinogenesis (Illeperuma et al., 2015). The study predominantly focused on the role of fibroblasts than keratinocytes. AN-extract increases tumor-promoting cytokines in the fibroblasts and these cytokines indirectly promote Reactive Oxygen Species (ROS) generation and DNA damage in keratinocytes, thereby leading to oral carcinogenesis. However, epithelial inflammation is also one of the histopathological features in OSF and is crucial for tissue fibrosis and carcinogenesis (Jeng et al., 2003).

Epithelial-to-mesenchymal transition (EMT) plays a crucial role in physiological and pathological processes like embryogenesis, wound healing, and cancer. In pathological aspects, it is a track for metastasis in epithelium-induced carcinoma. EMT process loses the cell-cell adhesion and gains the cellular motility, thereby transforming into invasive and circulating tumor cells (Barriere et al., 2015). Also, it activates differentiation of myofibroblasts and deposition of the extracellular cellular matrix (ECM) via TGF-β, Wnt-β-catenin, and Fibroblast activation protein (FAP), and plays a role in renal, pulmonary, cardiac, hepatic, and skin fibrosis (Stone et al., 2016).

Collectively, we aimed to investigate the direct influence of AN-extract in oral keratinocytes, especially in mediating EMT-like phenotypical changes, and to evaluate the therapeutic potentials of antioxidants in the epithelium, as well as the stroma.

## Materials and Methods

*Cell culture*

We used immortalized human oral keratinocytes (IHOK) obtained by human papilloma virus (HPV) 16 E6/E7 transfection (Lee et al., 2005). The cells were authenticated using STR analysis by Korean Cell Line Bank. The cells grow in F-medium, which consisted DMEM and Ham’s nutrient mixture F12 (Gibco BRL, Grand island, NY), at a 3:1 ratio, supplemented with 10% Fetal bovine serum. The cells were maintained in a humidified incubator at 37ºC and in atmosphere containing 5% CO_2_. The culture medium was changed every 3 days for subcultivation.

*Preparation of AN-extract*

Preparation of AN-extracts has been described previously (Illeperuma et al., 2015). In brief, dried ripe AN-extracts were weighed and dissolved in sterilized ice cold distilled water vortexing at 4ºC overnight and centrifuged at 5,000 rpm for 15 min. Thereby, we used the supernatant after filtering.

*Enzyme-linked immunosorbent assay(ELISA)*

Sandwich Enzyme-Linked Immunosorbent Assay (ELISA) was employed to measure cytokine levels in the conditioned media according to 30 µg/ml of AN-extract treatment for 24 h, based on previous study. All antibodies of ELISA were purchased from R&D systems (Mineapolis MN, USA) and described in detail (Illeperuma et al., 2015). Briefly, Gro-α (4μg/ml), IL-6 (2μg/ml) and IL-8 (8μg/ml) were captured on each well. After incubation for overnight, the supernatant was collected, centrifuged and filtered. To determine antioxidant effect in AN induced IL-6 secretion, the three type of antioxidants (Epigallocatechin-3-gallate (EGCG; 12.5 μM; DSM Nutritional Products Ltd, Basel, Switzerland), L-Glutathione-reduced (Glu; 5 mM; Sigma-aldrich, St. Louis MO, USA), N-acetyl-cysteine (NAC; 10 μM; Enzo Life Sciences, Farmingdale NY, USA)) were used. Cytokine secretions (pg/ml) were normalized according to total protein concentration (mg/ml)

*Protein Extract and Western Blot*

IHOK cells (1 × 10^6^) were harvested in Cell Lysis Buffer (Cell Signaling Technology, Danvers MA, USA), containing phenylmethylsulfonyl fluoride (PMSF) (Sigma-aldrich, St. Louis MO, USA). The lysates were incubated for 30 min on ice with vortexing every 5 min and, the samples were centrifuged at 15,000 rpm for 10 min at 4ºC to remove insoluble debris. After centrifuging, the supernatant was transferred to new micro-centrifuge tube and then mixed with 5X SDS sample buffer for 5 min at 95-100ºC. Proteins (50 μg) were loaded and separated on a SDS-PAGE and transferred on polyvinylidene difluoride membranes. Antibodies against E-cad (mouse mAb), Vimentin (Rabbit) and Snail (mouse mAb) were purchased from cell signaling technology. β-actin(Bioworld Technology, St. Louis Park MN, USA) was used as the housekeeping protein.

*Immunofluorescence*

To find out whether AN-induced IL-6 could induce epithelial-mesenchymal transition (EMT) in IHOK cells, direct immunofluorescent staining was carried out. Stabilized cells were seeded onto chamber slides (Nalge Nunc, Roskilde, Denmark) treated with IL-6 (50ng/ml). The cells were double-stained with cytokeratin and vimentin (FITC conjugated anti-cytokeratin (1:200) and Cy3 conjugated anti-vimentin (1:200) (Sigma-aldrich, St. Louis MO, USA). Lastly slides were stained with 10 μg/ml DAPI for 30 min, and then mounted. The slides were analyzed by confocal microscopy (LSM510 Meta, Carl Zeiss, Jena, Germany)

## Results

*AN-extracts induces IL-6 secretion in IHOK*

**Figure 1:**
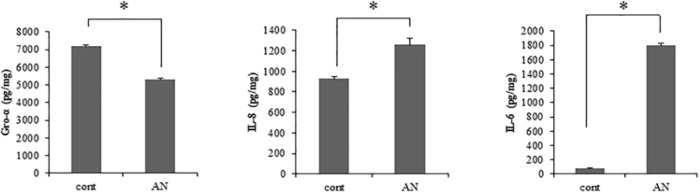
Areca Rut Extract Induces Tumor-Promoting Cytokines in IHOK Cells. IHOK cells were seeded onto 6 well. Areca nut (AN) extracts (30 µg/ml) were applied with serum-free media at overnight. The conditioned medium was collected. The supernatants were utilized for detecting each cytokine. (*p < 0.05 by Mann Whitney U test)

We previously demonstrated that AN-exposed gingival fibroblasts secrete various tumor promoting cytokines, including Gro-α, IL-6 and IL-8 (Illeperuma et al., 2015). We assessed whether AN-extract induces the cytokine secretion in oral epithelial cells as well as fibroblasts. Out of the three cytokines, IL-6 and IL-8 showed that 22.00- folds and 1.36-folds increase in secretion level, respectively, and Gro-α showed 1.35-folds decrease in the presence of AN-extract (Figure 1). Especially, IL-6 showed the highest increased ratio in AN-exposed IHOK, compared to the secreted level of IL-8. Subsequently, we focused on the IL-6 secreted by AN-extract. 

*IL-6 induces EMT in IHOK*

**Figure 2:**
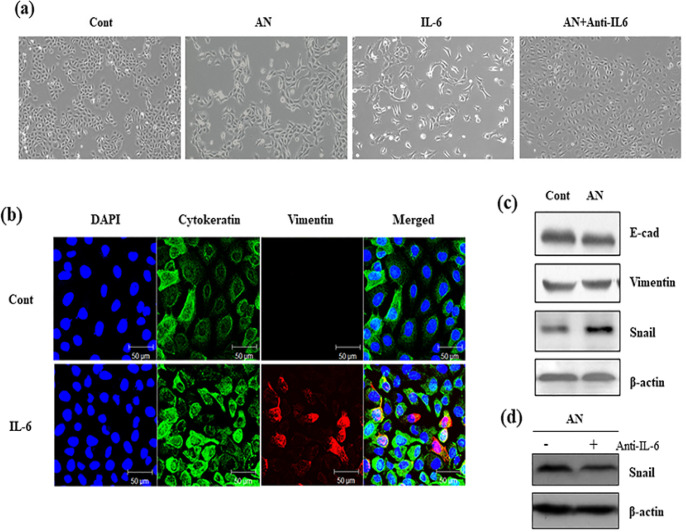
IL-6 Induces EMT-Like Phenotypes in IHOK Cells. (a) Morphological change was observed. AN(30 µg/ml), IL-6 (50 ng/ml) and Anti-IL6 (100 ng/ml) were applied to each wells for 48 hr. The magnification of all images is X200. (b) Representative immunofluorescent images were indicated. DAPI (blue), Cytokeratin (Green), Vimentin (Red) and Merged images were shown as the magnification X400. Scale bar is 50 µm. (c) AN(30 µg/ml) was treated to IHOK cells (1 x 106) for 24 hr, and then cells were extracted to detect the expression of EMT-related protein, E-cadherin (E-cad), Vimentin and Snail. Distilled water was applied as control cells. (d) IHOK cells (1 x 106) were applied AN (30 µg/ml) with or without Anti-IL6 (100 ng/ml). After 24 hr, cells were extracted

Next, we investigated the role of AN-induced* IL-6* expression in IHOK. IHOK was treated with AN-extract and IL-6, respectively, and changes in the cell morphology were analyzed (Figure 2A). We hypothesized that AN-induced* IL-6* expression could lead to phenotypical change, like EMT. To prove the hypothesis, IHOK were stained with cytokeratin and vimentin. With cell elongation, the cells increase the expression of Vimentin induced by IL-6 (Figure 2B). Furthermore, the EMT-related protein expressions were identified. Expectedly, AN-extract did not increase the expression of E-cadherin, and conversely, increased the expression of vimentin and snail (Figure 2C), indicating that AN-extract can induce EMT in IHOK. To verify that AN-induced EMT is due to IL-6, AN-extract was treated with neutralizing antibody of IL-6 in IHOK. Although AN-extract was applied in IHOK, AN-induced snail expression was reversed by neutralizing IL-6 (Figure 2D). Taken together, it is evident that AN-extract can induce IL-6 expression, thereby leading to EMT. 

*Antioxidants prevent IL-6 secretion by AN-extract*

**Figure 3:**
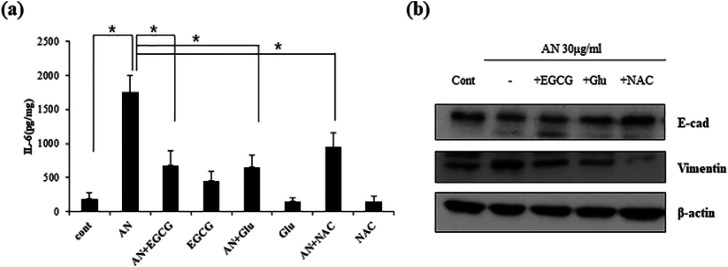
Antioxidants Block AN-Extract Induced IL-6 in IHOK Cells. (a) Three antioxidants (EGCG; 12.5 µM, Glu; 5mM, NAC; 10 µM) were treated with or without AN(30 µg/ml) in serum-free media. After 24 hr, conditioned medium was collected. The supernatants were utilized to detect IL-6. (*p < 0.05 by t test). (b) Cells (1 x 10^6^) were seeded onto 100 mm dishes. Antioxidants (EGCG; 12.5 µM, Glu; 5 mM, NAC; 10 µM) were treated with or without AN (30 µg/ml) in serum-free media. The protein was used to identify EMT-related proteins

Previously, we suggested that antioxidants might have therapeutic effects in patients with oral diseases caused due to the habit of chewing AN, based on the observation that ROS-induced DNA damage in oral keratinocytes and ROS generation stimulated by secreted cytokines in fibroblasts were blocked by antioxidants, such as EGCG, glutathione and NAC (Illeperuma et al., 2015). Hence, we investigated the role of antioxidants in AN and IL-6 induced cellular responses. First, the capability of antioxidants was examined in blocking IL-6 secretion (Figure 3A). After treatment with the three types of antioxidants, AN-induced IL-6 secretion was significantly reduced.

 Based on these results, we additionally identified EMT-related protein expressions (Figure 3B). As a result, AN-decreased E-cadherin was reversely increased by antioxidants. Similarly, AN-increased vimentin was reversely reduced by antioxidants. Out of the three antioxidants, NAC treatment induced down-regulation of vimentin expression and up-regulation of E-cadherin in the most effective manner. No significant difference in the expression of snail was found between AN-treated cells and antioxidants-treated cells (data not shown). These findings imply that antioxidants diminish during AN-induced EMT process, indicating that antioxidants might partially prevent AN-induced carcinogenesis.

## Discussion

OSF is an oral potentially malignant disease caused by AN chewing (Tilakaratne et al., 2006; Arakeri et al., 2017). The fibrotic reaction of OSF has the potential to promote malignancy in the neighboring epithelial cells and create the tumor microenvironment through the crosstalk between fibroblast and epithelial cells(Jung et al., 2010; Bae et al., 2014). Based on these pathological findings, we previously demonstrated that AN-exposed fibroblasts play an active role in oral carcinogenesis by producing tumor-promoting cytokines. As a characteristic feature of OSF, epithelial atrophy is also a pathognomonic finding (Khan et al., 2015) and, in turn, cancer arises from epithelial transformation. Thus, the present study aimed to investigate AN-induced EMT in epithelial cells in terms of cytokine secretion. 

AN induces the generation of ROS and then DNA damage in oral epithelial cells (Illeperuma et al., 2015). It is associated with precancerous epithelial transformation and epithelial atrophy (Farinati et al., 1998; Khan et al., 2015). In addition, AN can initiate multiple oncogenic signaling pathways, such as JNK (Pant et al., 2016), NF-κB and MAPK (Lin et al., 2005), thereby, eliciting the inflammatory response by mediating the secretion of growth factors and cytokines, including TGF-β, IL-1α, IL-8, and IL-6 (Chang et al., 2009; Illeperuma et al., 2015; Chang et al., 2016; Pant et al., 2016). In particular, IL-6 is a key mediator in inflammation and carcinogenesis, such as EMT, metastasis and tumor microenvironment (Scheller et al., 2006; Yadav et al., 2011; Son et al., 2015). As IL-6 is secreted in both AN-exposed fibroblasts and epithelial cells, its blockade could prevent tumor microenvironment, which is required for malignant transformation of epithelial cells and fibroblasts.

EMT has conflicting features as a double-edged sword: such as organogenesis, wound healing and cancer development (Barriere et al., 2015). However, AN-induced EMT has been studied in cancer progression, rather than in normal physiological processes. Arecoline, the component of AN-extract, can induce EMT, thereby exacerbating cancer progression (Zheng et al., 2018; Chang et al., 2019; Yao et al., 2019). Thus, AN-induced EMT is implicated in the primary mechanisms by which epithelial cancer cells acquire malignant phenotypes that promote metastasis. Our results showed that AN-extract induces EMT-like phenotype, such as morphological changes and snail protein upregulation. Consistently, proteomics analysis has reported that EMT as the most prominent pathway in AN-treated oral cancer cells (Chiang et al., 2016). For instance, AN-treated cells increase invasive ability along with EMT (Chiang et al., 2016; Li et al., 2016). In this study, it was additionally demonstrated that IL-6 is a key mediator of EMT in AN-treated oral keratinocytes. 

Antioxidants protect the cells from damage caused by free radicals containing ROS, which can promote cancer initiation and progression through aberrant cell signaling pathways that regulate cellular proliferation, survival, angiogenesis and metastasis (Glasauer and Chandel, 2014). In this study, three types of antioxidants, including EGCG, Glutathione and NAC were employed. The antioxidants demonstrated reversal of AN-induced EMT by inhibiting IL-6 secretion, suggesting that they could block AN-induced EMT. EGCG is one of the most important components of the green tea catechins and possesses anti-cancer properties; it influences many signal transduction pathways, such as JAK/STAT, MAPK, PI3K/AKT and NF-κB (Singh et al., 2011). Similar to other antioxidants, glutathione is largely known to interrupt lipid peroxidation of cell membranes and oxidative stress, and NAC is an antioxidant supplement that is used to maintain optimal glutathione level (Kerksick and Willoughby, 2005). Both the antioxidants mediate the NF-κB pathway. Concerning the signaling pathways involved in the anti-cancerous activity of the antioxidants, we already demonstrated that AN-induced tumor promoting cytokines were blocked by antioxidants and their cytokines was mediated by NF-κB in the fibroblasts (Illeperuma et al., 2015). Therefore, it was presumed that the antioxidants can impede AN-induced cellular responses by the NF-κB signaling pathway in oral epithelial cells. Moreover, it has been reported that NAC and EGCG inhibited ROS induced snail expression caused by arecoline, which is one of the key components of AN-extract (Lee et al., 2013). Correspondingly, our data showed that AN-induced EMT was reversed by antioxidants. Additionally, we suggested that IL-6 should be an initiator for AN-induced EMT, and antioxidants prevent AN-induced EMT by inhibiting IL-6 secretion. Taken together, it was revealed that antioxidants are very effective against EMT in AN-treated epithelial cells, as well as in oral carcinogenesis, thereby indicating that antioxidants might be powerful therapeutic agents to prevent malignant transformation of OSF into OSCC. 

In summary, this study demonstrated that AN-extract induced IL-6 secretion in epithelial cells, thereby mediating EMT. These processes were interrupted by antioxidants. In conclusion, our study might facilitate further studies for investigating the transformation mechanism of OSF into OSCC highlights antioxidants as a prototype for the development of therapeutic interventions in OSF. 
